# Soccer Scoring Techniques—A Biomechanical Re-Conception of Time and Space for Innovations in Soccer Research and Coaching

**DOI:** 10.3390/bioengineering9080333

**Published:** 2022-07-23

**Authors:** Gongbing Shan, Xiang Zhang

**Affiliations:** 1 Biomechanics Lab, Faculty of Arts & Science, University of Lethbridge, Lethbridge, AB T1K 3M4, Canada; 2 Department of Physical Education, Xinzhou Teachers’ University, Xinzhou 034000, China; zhangxiang@xztu.edu.cn

**Keywords:** proprioceptive shooting volume, zero possession shot, scoring opportunity identification, airborne, biomechanical modeling, anthropometry

## Abstract

Background: Scientifically, both temporal and spatial variables must be examined when developing programs for training various soccer scoring techniques (SSTs). Unfortunately, previous studies on soccer goals have overwhelmingly focused on the development of goal-scoring opportunities or game analysis in elite soccer, leaving the consideration of player-centered temporal-spatial aspects of SSTs mostly neglected. Consequently, there is a scientific gap in the current scoring-opportunity identification and a dearth of scientific concepts for developing SST training in elite soccer. Objectives: This study aims to bridge the gap by introducing effective/proprioceptive shooting volume and a temporal aspect linked to this volume. Method: the SSTs found in FIFA Puskás Award (132 nominated goals between 2009 and 2021) were quantified by using biomechanical modeling and anthropometry. Results: This study found that players’ effective/proprioceptive shooting volume could be sevenfold that of normal practice in current coaching. Conclusion: The overlooked SSTs in research and training practice are commonly airborne and/or acrobatic, which are perceived as high-risk and low-reward. Relying on athletes’ talent to improvise on these complex skills can hardly be considered a viable learning/training strategy. Future research should focus on developing player-centered temporal-spatial SST training to help demystify the effectiveness of proprioceptive shooting volume and increase scoring opportunities in soccer.

## 1. Introduction

Soccer is the most popular sport in the World. Based on the information from Fédération Internationale de Football Association (FIFA), the game is played and watched on five continents with 265 million players and 4 billion fans, i.e., over 50% of the world population (7.7 billion) are linked to the game [1,2,3]. Yet, contrary to the popularity of the game, the number of scientific inquiries on key motor control skills, i.e., soccer scoring techniques (SSTs), appears disproportionately low when compared to the participation-to-scientific study ratios of other sports skills, such as complex gymnastics skills [4]. As a result, the scientific understanding of SSTs lags far behind its practice, with most participants acquiring various SSTs through individual experience rather than science-based instruction [5,6]. To make the matter worse, there is a dearth of scientific investigation on the many complex SSTs, such as the jumping turning kick, e.g., a nominated goal for the FIFA Puskás Award 2019, performed by Ibrahimović [7] and the diving scorpion kick, which won the FIFA Puskás Award 2017 [8]. These SSTs seem virtuosic in scope and are commonly believed to be the talent ability solely of soccer stars [9]. Obviously, relying on the aptness of the athletes to improve these virtuosic SSTs can hardly be considered a viable learning or coaching strategy. Worst of all, the terms surrounding SSTs used in practice and research have been confusing [10]; researchers and practitioners are not sure how many SSTs are available for the game. This scenario hinders not only the scientific studies on many exceptional SSTs but also the development of novel coaching methods for learning these SSTs.

It is well known that the greatest attraction of soccer is goal scoring. Compared to many other sports, goals are relatively rare in soccer–on average, less than three goals per game in FIFA world cups since the 1960s [11]. Because of their rarity, soccer goals are extremely exciting for millions of fans. The various means by which gameplay moves toward a goal can be thought of as an improvised drama, where emotional tension is built over long periods only to be fully released when the goal is achieved. This characteristic contributes to making soccer the most popular spectator sport in the world. Therefore, an essential core of soccer research and coaching is to help athletes master aesthetically eye-catching SSTs for increasing both scoring possibilities and the excitement of the game.

### 1.1. Novel Scientific Studies Required for the World’s Most-Popular Sport

Through systematic identification, a recent study [10] has revealed that there are 43 SSTs that exist in current soccer games. Surprisingly, there are only a handful of SSTs documented in the existing training/coaching literature and, consequently, the comprehensive list of SST training is presently limited to maximal instep kick (including the curled kick), jumping headers, and volley/side volleys [12,13,14]. Since the existing scientific understanding could not supply enough help to practitioners in developing training methods for learning more SSTs, practical *modi operandi* have been the main driving force for keeping SST exuberant.

At the practical level, celebrations of the goal of the month, of the season, of the year, and of the decade, are widely adopted in professional soccer leagues for recognizing players who are deemed to have scored the “most beautiful goal” [10]. Those glorious goals are normally chosen by a combination of panel experts and a public vote. This practice has been successfully used to encourage and promote aesthetically significant goals from players, as it sparks the creativity of the athletes to develop novel/unique SSTs through “self-learning”. Even infrequently, under this practical *modi operandi*, exceptional SSTs have been developed by a few talented athletes from time to time. These virtuosic skills have become models for more athletes and coaches to impersonate and duplicate blindly, i.e., learning without insight knowledge obtained from research. Especially the brilliant goals scored in soccer’s flagship tournaments, such as those held by FIFA, UEFA (Union of European Football Associations) and other professional national soccer leagues with an international reputation (e.g., La Liga/Spain, Serie A/Italy, Bundesliga/Germany, Premier League/England, and Ligue 1/French), are mimicked by worldwide soccer players. Biomechanically, there are two issues related to such blind self-learning: learning efficiency and injury risk.

Soccer shots, like many complicated human movements, are trained motor skills. Studies have demonstrated that systematic and scientific inquiry into the biomechanics of human motor skills has great potential to demystify complicated human motor skills for efficient learning [15,16,17,18]. Hence, knowledge obtained from biomechanics studies can help optimize performance outcomes while simultaneously reducing the risk of training-related injury. There are two useful biomechanical analyses: kinematic and kinetic quantification of motor skills. In terms of motor learning, kinematics has significant utility in terms of improving teaching and learning, while an understanding of kinetics is essential for reducing the risk of learning and playing-related injury. Both kinematics and kinetics have significant utility in raising practitioners’ awareness of biomechanical “cause and effect”, thus giving them additional tools and knowledge to optimize their practice [6,17,19,20,21].

From a motor learning point of view, one common criterion of the honored goals in soccer’s flagship tournaments is their repeatability, e.g., the FIFA Puskás Award states that “the goal should not be the result of luck or mistakes by the other team” [22], suggesting that the nominated goals and the related SSTs are theoretically repeatable, i.e., entrainable. What is needed for establishing a scientific training system is the knowledge of the biomechanical parameters that influence the quality of the soccer shots. Unfortunately, most of the 43 SSTs [10] identified so far are “off the radar” to researchers. The SSTs overlooked in research are normally airborne and/or acrobatic, perceived as high-risk and low-reward. Counting on athletes’ talents to improvise on those SSTs is neither scientific nor realistic. Hence, novel biomechanical studies are needed for the scientific discovery of these airborne and/or acrobatic SSTs. As a result, the skills that are virtuosic in appearance may be eminently trainable and less a product of the improvisatory abilities of individual players.

### 1.2. Literature Review

Web of Science is a reputable resource due to its guaranteed proofed scientific content [23]. Among all types of papers, review articles draw upon published articles to provide a great overview of the existing literature on a topic. As such, to reveal the current state of scientific studies on soccer scoring with rigorous and quality information, the Web of Science database was searched for systematic review articles in December 2021. The search was performed by using the keywords “football” and “soccer“, with each associated with the terms “goal analysis” and “review”. The initial search identified 31 papers in the database. The titles and abstracts of 31 articles were then screened according to their relevance to SSTs in elite soccer, resulting in 27 studies being eliminated. At the end of the screening procedure, four systematic review articles [24,25,26,27] between February 2018 and January 2020 received further in-depth reading to identify the current research state in this area. The articles show that soccer scoring quantification started as early as 1968, investigating the statistical relationship between the number of passes and goals scored by analyzing 3213 professional matches from 1953 to 1968 [28]. Over the past half-century, more and more notational measurements were developed and applied to finding the factors influencing soccer goal scoring. These measurements include total passing frequencies [29], ball possession time [30,31], shots at goal [32,33], the position of an attempt on goal (e.g., three longitudinal areas: right, center, and left, and zone: ultra-defensive, defensive, central, offensive, and ultra-offensive) [34,35], the influence of set plays or dead ball routines [36,37] etc. In short, the previous studies on soccer goals have overwhelmingly focused on the development of goal-scoring opportunities or game performance analysis, i.e., tactical strategies, and overlooked the role of various SSTs on soccer goals.

As matter of a fact, very few studies have analyzed the special kicking skills that elite goal scorers possess [9,27]. As revealed by the recent systematic review article, the existing research studies have not fully explored the final actions of the players in goal situations [27]. There is no doubt that the current studies can provide important references for coaches in designing training programs for developing the tactical strategies of a team. However, they could not improve the SST training. It is well known that all tactical strategies learned and trained aim at achieving the ultimate shot opportunity, and the key to the success or failure of the shot depends largely on the SST selected by the player.

A study on defense in all 306 German Bundesliga games from the 2010/2011 season [38] showed that the top teams have a faster defensive reaction time compared to the remaining teams. The result suggests that the time taken to perform a shot is becoming shorter and shorter as the competition level increases. FIFA has vividly described this development trend as “every nanosecond is special” [39]. As a consequence, more and more airborne shots are seen in current elite games, and the height of an airborne kick is increasing higher and higher in the top elite-level games. Unfortunately, both current research and existing training systems can no longer keep up with actual real-game development.

### 1.3. The Current State of SST Knowledge

In this vein, few existing studies have found that shots with the feet seem to achieve between 70–80% of the goals, whereas the rest of the goals are achieved from headers [40,41]. More details have been added by a recent study [10], finding that there are 43 SSTs identified in elite soccer games, and over 60% of them are airborne SSTs. The airborne shots contribute to over 50% of goals, with shots attempted by foot, head, or chest. These findings indicate that scoring opportunity identification has to consider factors linked to airborne shots. Sadly, the most up-to-date systematic review article on the biomechanics of kicking in soccer is a dozen-years old, which focuses only on the instep kick [42], with a dearth of scientific studies on most of the airborne SSTs [4,43].

There are also limited studies on the anatomic parts of the foot used to shoot. One study has found that the instep seems to be the most used anatomic part, followed by the inside part of the foot [44]. The recent study conducted by Zhang and Shan [10] added more details to this; depending on the scorer’s body posture when shooting (e.g., facing, side-facing, or back-facing) and ball position (e.g., ground or airborne), the instep, dorsi-side, inside, outside, toe, heel, or plantar-side are selected by scorers for shots.

Due to the limited knowledge related to SSTs, there is a scientific gap in current scoring-opportunity identification. From a scientific standpoint, both temporal and spatial variables must be examined when evaluating scoring opportunities. In essence, scoring chances are an issue of maximizing the probabilities of scoring, which cannot be determined without basic theories and knowledge related to the temporal-spatial identification and quantification of a player’s possibilities to shoot.

From the temporal perspective, it is well known that even if in possession of a free ball, a player will likely not be free for long; defenders will attempt to thwart the shot. A representative study [40], which analyzed all goals in the English Premier League during the 2008/2009 season, found that scoring with zero possession (i.e., a “one-touch shot” where a player shoots as a ball is passing by) accounts for 69.3% of goals. Setting up a shot with one or more contacts of the ball results in only 17.9% of goals. These results indicate that the longer a player possesses the ball, the lower the scoring chance. The least favorable situation for scoring is when players score after individual dribbling (12.8%).

Regarding spatial variables, existing studies have shown that 65–90% of goals are scored when an attacking player possesses the ball in or near the opponents’ penalty area, and he/she is not hindered by defenders, i.e., a “free ball” [44,45,46,47]. The studies have actually confirmed the practitioners’ empirical evidence. This explains why most of the current goal-scoring training overwhelmingly emphasizes the geographic location of the ball on the playing field.

In brief, the present research scheme fails to consider the player-centered temporal-spatial aspects of SSTs and underemphasizes air-attack (3D consideration) related to shooting techniques.

### 1.4. Research Aims and Research Questions

The current scenario would suggest that ground-breaking research is needed in order to develop science-based SST training regimes for improving scoring possibility. The current paper aims to lay a foundation for launching a ground-breaking study via the re-conception of temporal and spatial factors related to soccer shooting, identifying elements that could be applied in the entrainment of complex SSTs via biomechanical quantification. The study has three specific research questions to be answered (i.e., the main goals of the current study):

- Scientifically, what is the content of temporal–spatial opportunity identification related to SST?
- What is a quantitative and reliable method to evaluate the improvement of scoring possibility through science-based training?
- What is the theoretical implication of these findings related to the innovative development of SST training?

## 2. Materials and Methods

For answering these research questions, the current study initiates a novel theoretical framework, which has its origin in elite soccer. Three steps are involved in the establishment of the original framework: (1) selection of temporal criteria for efficiency recognition, (2) identification of new/potential spatial variables via the video-based analysis of all 132 nominated goals of the FIFA Puskás Award between 2009 and 2021 [22], and (3) quantification of scoring possibility via biomechanical modeling.

### 2.1. A New Theoretical Framework for Developing SST Training—Focusing on Time in Space

Scoring opportunity can be defined by the feasibility of shooting successfully. Practically, it is well known that when an attacker gets a scoring opportunity, his/her chance to shoot the ball typically does not last very long. In these brief moments, a player needs to shoot the ball quickly (the temporal aspect of goal scoring) and accurately (the spatial feature of goal scoring). The reality in elite games is actually more complicated than this simplified thinking. Regardless of the geographic opportunities one could gain, only considering the dynamic relationship between an attacker, the goal, and the 3D position of the ball related to the scoring chance identification, is not enough to reach a decision without knowing the biomechanical characteristics of various SSTs. Therefore, a new framework should link the temporal and spatial details to the SSTs for ameliorating the scoring possibility.

Since there is no existing framework for considering the temporal and spatial aspects of SSTs simultaneously, our study will bridge the gap by creating a new theoretical framework. The original framework covers factors related to the temporal efficiency and spatial effectiveness of SSTs. The basic ideas of the framework are: (1) to mathematically evaluate the effective shooting volume for scoring chance quantification, i.e., a quantitative determination of spatial feasibility for shooting, and (2) for any dynamic ball (chance) covered by the effective shooting volume, to choose a proper SST for shooting without delay, i.e., temporal efficiency of shooting. The novel aspect of the original framework is to introduce 3D considerations (of player-centered temporal-spatial consideration) for the training of SSTs in order to improve scoring possibility.

The key element of the framework is the effective shooting volume. Its quantification will inevitably require the study of the overlooked airborne and/or acrobatic SSTs. These SSTs are intricate, belonging to gymnastic-like motor skills. Previous studies have revealed that the learning quality of gymnastic-like motor skills depends on one’s proprioceptive ability and can be progressively developed through structured repetitive training [48,49,50,51]. Hence the effective shooting volume can also be named as the player’s proprioceptive shooting volume. This volume determination would fundamentally inform if a dynamic ball should be counted as a goal chance (i.e., the ball falls within or passes through the volume) or not.

In summary, the core of the novel framework is to create a new quantitative way for (1) enlarging the effective shooting volume to cover more goal chances and (2), at the same time, enhancing the shooting ability to ensure one-touch shots within this volume. Based on these core elements, this original framework can be denominated as “Focusing On Time In Space”. It aims to nexus the temporal efficiency and spatial effectiveness of maximizing soccer scoring possibilities. Whence, it would build a scientific foundation for innovations in future SST research and training.

### 2.2. Selection of Temporal Criteria

Our selection of temporal criteria is based on a study related to the temporal efficiency of SSTs carried out by Durlik and Bieniek (2014) [40]. Since players scoring after individual dribbling involves other motor skills rather than SST skills only, a modification has been made, i.e., the scoring after individual dribbling was excluded. Additionally, any further actions before shooting have been proven to decrease scoring possibility [40]; therefore, it is logical to select the amount of possession before shooting so as to recognize overall temporal efficiency. Hence, the following categories were selected for the quantification in this study:

- one-touch-shot (zero-possession), where a player shoots as a ball is passing by;
- One possession, i.e., setting the ball and then shooting;
- the other ball control strategies combined, i.e., 2+ possession maneuvers.

The temporal criteria were evaluated via the statistical results of the 132 Puskás nominated goals between 2009 and 2021 [22].

### 2.3. Identification of New Spatial Variables

**Figure 1:**
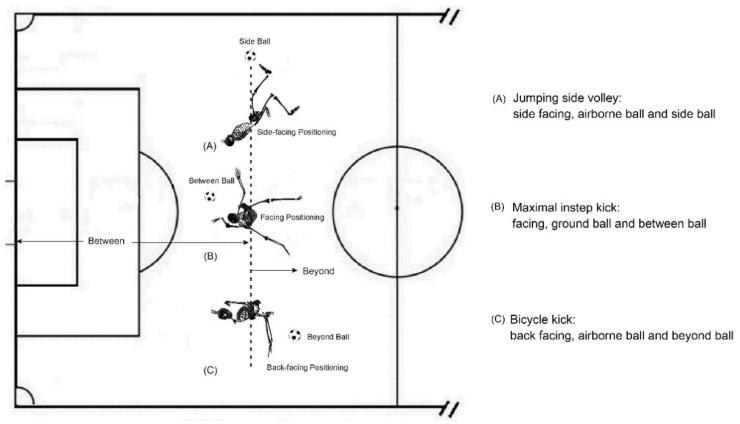
The identification and clarification of spatial factors. (**A**) The 3D quantification of a jumping side volley–top view [4,43]. (**B**) The 3D quantification of the maximal instep kick–top view [52,53,54]. (**C**) The 3D quantification of the bicycle kick–top view [9,43].

Regarding spatial analysis, previous studies have focused only on field geography [44,45,46,47] and have neglected those factors related to SSTs, e.g., the athlete’s body orientation facing, side-facing, or back-facing the goal, and the spatial position of the ball at the instance of a shot [10]. To give more detail to the latter aspect, the spatial position of the ball has horizontal and vertical components. Horizontally, using the goal and the player as positional references, the ball can be between them, beyond them, or to the side of them. Vertically, the ball can be airborne or on the ground (Figure 1). Therefore, in the current study, these new spatial parameters related to SSTs at the instance of a shot were induced and designated for quantitative analysis.

Combinatorically, the above selected temporal and spatial parameters were applied to the quantitative examination of all 132 Puskás shooting videos. Descriptive statistics (i.e., pie chart) were applied to summarize these categorical data (i.e., percentage distribution) in order to reveal the characteristics of the selected parameters and their contributions to scoring goals at the top elite level.

### 2.4. Quantification of the Proprioceptive Shooting Volume via Biomechanical Modeling

Dimensional data obtained from 3D motion capture and/or full-body biomechanical modeling were used to estimate the proprioceptive/effective shooting volume. The quantification works as follows:

- Apply 3D biomechanical modeling to quantify various kicking techniques [4,9,53,55];
- Target and select three SSTs to determine the anterior–posterior, medial–lateral, and vertical dimensions when the kicks are being performed. The selection of SSTs represents the shooting ability that an athlete can obtain after the current training methods or that talented elite players have already performed in elite soccer;
- Use the three dimensions of the selected SSTs to estimate the proprioceptive shooting volumes of the two different performance levels and to determine the difference between them.

The 3D dimensional data used in this study are either from the authors’ previous 3D quantification studies or gained through both anthropometrical study [56] and biomechanical model estimation/simulation. Clearly, different selections of SSTs will result in distinct sizes of the volume, i.e., various SSTs will lead to a differentiation in scoring possibility. Logically, the larger one’s effective shooting volume, the more goal chances one will possess. Through the novel framework, the current article has, for the first time, visualized the concept of how to establish a link between the temporal-spatial aspects of SSTs and goal chance quantification. This conceptual exploration is currently missing in soccer research and practice.

## 3. Results

**Figure 2:**
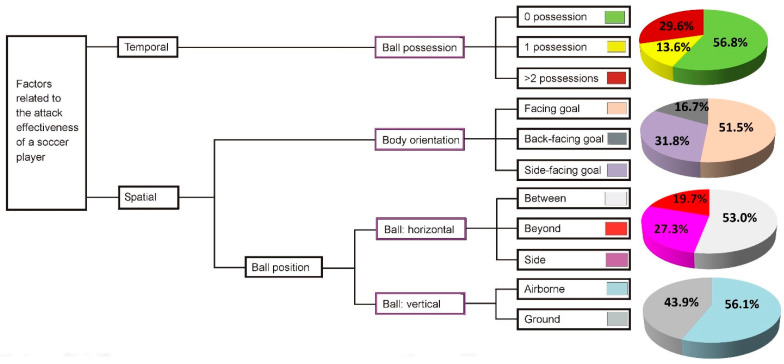
The temporal and spatial factors related to goal chance identification and their contributions to goals: evidence drawn from all 132 FIFA Puskás Award nominees’ goals, 2009–2021.

The analysis results of temporal and spatial factors related to goal-chance identification are shown in Figure 2. Compared to the outcomes of temporal efficiency from the previous study [40], i.e., 69.3% of goals scored for zero-possession shots, 17.9% of goals scored after two or more possessions, and 12.8% of goals scored for shots after dribbling, the temporal branch of Figure 1 based on the 132 FIFA Puskás Award nominated goals shows comparable results: 56.8% (0 possession), 70.5% (0 & 1 possession), and 29.5% (2 + possessions), respectively.

The results of the spatial analysis of the 132 Puskás goals denoted that, as a ball comes into an athlete’s proprioceptive shooting volume, a player’s body orientation can be facing, side-facing, or back-facing (Figure 1 and Figure 2) relative to the position of the goal. In terms of body orientation, at the moment of shooting among the 132 Puskás goals, about half (51.5%) were achieved facing the goal, while 31.8% were “side-facing”, and 16.7% were “back-facing” (Figure 2). The statistics for horizontal ball position are comparable, with 53.0% of goals having occurred when the ball was between the player and goal, while side and beyond balls accounted for the remaining 47.0%. Regarding vertical ball positioning, 56.1% of the goals were airborne, and only 43.9% were ground balls.

**Figure 3:**
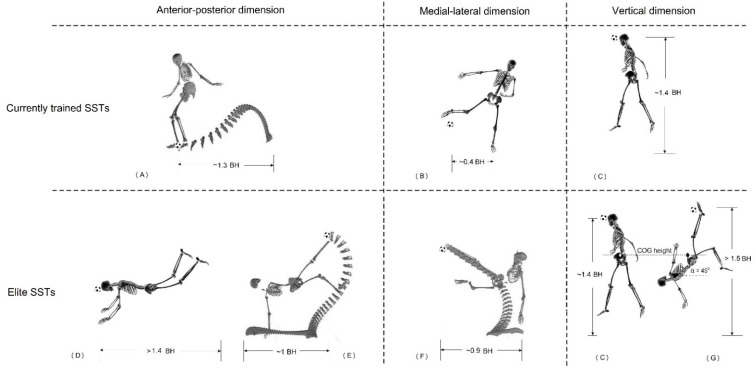
The dimensions of selected SSTs and their influences on the quantification of the attack space (scoring chance): (**A**) The 3D quantification of the maximal instep kick–side view with timely trace of the kicking foot, obtained via 3D-motion analysis and biomechanical modeling [52,53,54]. (**B**) Lateral dimension of volley kick–distance estimated using biomechanical modeling and anthropometrical study [5,56]. (**C**) Vertical dimension of a jumping header–distance estimated using jump biomechanics [57,58] and anthropometrical modeling [56]. (**D**) Frontal dimension of a diving header–distance estimated using diving biomechanics [59] and anthropometrical modeling [56]. (**E**) The 3D quantification of the bicycle kick–side view with timely trace of the kicking foot, obtained via 3D-motion analysis and biomechanical modeling [9,43,56]. (**F**) The 3D quantification of jumping side volley–frontal view with timely trace of the kicking foot, obtained via 3D-motion analysis and biomechanical modeling [4,43]. (**G**) Potential attack height reached by using a bicycle kick at different trunk-angle orientations–biomechanical model estimation [9,56].

The three-dimension comparisons between the SSTs trained by the current system and the selected SSTs performed by elite players are shown in Figure 3. As elaborated in the method section, these three-dimensional values were applied to quantitatively estimate the shooting volume formed by skills in terms of a player’s body height (BH). It should be noted that (1) the estimation represents the maximum volume one could reach, and (2) the dimensions normalized by BH increased the generalization of the method [55]. In current soccer coaching practice, the systematically trained SSTs are the maximal instep kick (including the curled kick), jumping headers, and volleys and side volleys [12,13,14]. Therefore, the proprioceptive shooting volume of the currently-trained SSTs could be calculated by the following dimensions:

- Anterior–posterior dimension is 1.3 BH, i.e., the last-stride length of the maximal instep kick [52,54,55] (Figure 3A);
- Medial–lateral dimension is 0.8 BH, i.e., the lateral reach of the side volley of 0.4 BH by each leg (Figure 3B);
- Vertical dimension of 1.4 BH, i.e., the jumping height for a header (Figure 3C).

In the case of a 1.8 m tall athlete, when the above dimensions are considered, the calculation of his/her maximum effective shooting volume would be 2.34 m × 2.52 m × 1.44 m, or very roughly 8.5 m^3^.

**Figure 4:**
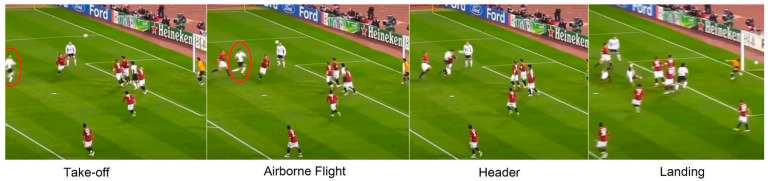
The long-jump header (the player is identified in red circle on the two left frames). Currently, there is no study available for revealing the 3D dimension of this SST (the figure was generated from the video of UEFA TV video, published in 2021 [60]).

A similar quantitative estimation was also applied to the selected SSTs performed by elite players. The practical potential of the quantification of the proprioceptive/effective shooting volume is shown in Table 1. For the same case (1.8 m tall player), if the volumes of two acrobatic SSTs (i.e., bicycle kick and jumping side volley, Figure 3E,F, performed by talented elite soccer players) are included in the calculation, the same player’s effective shooting volume would be 4.14 m × 2.52 m × 3.24 m, roughly 35.4 m^3^, or approximately four times the shooting volume of the normally practiced techniques in current training practice. If additional SSTs, such as the diving header (Figure 3D) and an improved bicycle kick (trunk angled 45°, in addition to the current 0° bicycle kick, Figure 3G) are included, the effective shooting volume increases to 4.32 m × 2.70 m × 5.04 m, roughly 58.8 m^3^, or approximately seven times the normally practiced shooting volume. Ad hoc, more quantitative estimations could be performed, e.g., the long-jump header [10] performed by Cristiano Ronaldo in the 2008/2009 UCL season (Figure 4). If this SST could be quantified, the effective shooting volume would definitely increase further.

## 4. Discussion

Due to the rarity of goal chances in soccer, it is highly relevant to establish a theoretical system for innovating research and training practice around goal scoring. Obviously, the re-conception of scoring chance quantification and its relationship to the temporal and spatial aspects of SSTs would have great potential to form a foundation on which novel developments of various SSTs could be built. Unfortunately, scientific studies on the influence of temporal-spatial factors on shooting effectively (i.e., quantify the scoring chance and turning that scoring chance into a goal) have not yet been conducted. This is why our study proposed a novel framework for this area. Based on the results, this section makes an effort to answer research questions 1–3 to elaborate the conceptual metaphors of the novel framework.

### 4.1. Temporal-Spatial Opportunity Identification

The evidence gained from the 132 FIFA Puskás goals has clearly indicated that a new theoretical framework is needed for re-examining and/or allocating the known and unknown factors related to existing SST, and breakthrough studies are desirable to quantify the influences of those factors on temporal-spatial opportunity identification.

#### 4.1.1. Temporal Efficiency

The first challenge would be a temporal enumeration, i.e., the number of possessions of the ball during preparation for shooting. As portrayed before, even if in possession of a “free ball”, a player will likely not be free for long; defenders will attempt to thwart the shot. Therefore, any delay in preparing a shot will decrease temporal efficiency. The results summarized in this study clearly indicate that a sudden attack would create the highest temporal efficiency for converting a scoring chance into a goal. This result is comparable to the representative study [40] on the analysis of all goals from the English Premier League during the 2008/2009 season. Yet, the temporal aspect does not stand alone, and obviously, it interacts with spatial factors.

#### 4.1.2. Spatial Effectiveness

The special aspect presents a more challenging concept, but one thing is certain: it must consist of more than mere field geography. A player’s proprioceptive abilities influence goal-opportunity identification, i.e., as the soccer ball comes into a player’s proprioceptive shooting volume, his/her ability to perform a shot under various body orientations with the 3D spatial positions of the ball must fall within these abilities in order for him/her to attempt to score. Current knowledge shows that proprioceptive abilities can be developed through repetitive training through structured and targeted training [48,49,50,51].

Yet, spatial factors may be over-simplified in the present training practice. There seems to be an existing research and coaching emphasis on shots taken (1) facing the goal and (2) with the ball between the player and the goal [12,13,14]. It is not surprising that most training and practice regimes concentrate on the variables that account for the higher percentages of goals found in the pie charts of Figure 2. However, this neglects a significant percentage of the scoring opportunities that may exist in smaller “slices of the pie”. Some of these might be improved through scientifically structured training. For example, Swedish player Ibrahimovic’s bicycle kick against England in 2013 (i.e., the Puskás award 2013 [61]) was a goal achieved as he ran away from, and not toward, the goal. This shot can be characterized as follows: 0 possession, back facing the goal, a beyond ball, and an air attack. Currently, there is no science-based training program available to practitioners for learning this skill [9,43]. Therefore, the skill is generally considered a product of the improvisatory and virtuosic abilities of an individual player, not a trainable one. The question arises whether or not the training regimes that develop the SST shooting components required for such “virtuosity” might improve scoring percentage by increasing a player’s proprioceptive/effective shooting volume. The 132 Puskás goals have shown that 16.7% of the goals were scored by using back-facing goal techniques, and 19.7% of the goals were scored with the ball located beyond (not between) the goal and the players (Figure 2). These “unusual” goals would signify that what is required is a new theoretical framework to logically link the temporal and spatial factors for a systematical exploration of these acrobatic skills for developing science-based training methods.

#### 4.1.3. Lost in Time and Space in the Current Scoring Research

The current study reveals that joint consideration of the temporal and spatial aspects of scoring has been mostly neglected in the current SST research and coaching literature, i.e., scientific studies on SST could be considered lost in time and space. The results of Puskás goals imply that a few talented elite athletes, via years of practice, have magically linked temporal efficiency and spatial effectiveness in developing improvisatory abilities. Such abilities can turn “impossible” goals (commonly identified as non-chance goals) into goals. The scientific re-conception considered in this study would suggest that the biomechanical quantification of the temporal–spatial factors of these extraordinary SSTs could help us demystify these “magic kicks”, and evidently identify a scoring chance as “yes” or “no” instead of “impossible”. Without the scientific quantification of these temporal–spatial challenges, the identification of a scoring chances is vague, subjective, and unclear. Unfortunately, this is the situation for the current studies on soccer scoring, especially for the spatial challenges related to SSTs.

In summary, the current study is the one that has attempted to originate a novel framework for scientifically pinpointing the content of temporal–spatial opportunity identification. As such, future innovations in SST research and training system development could be launched by applying the proposed framework.

### 4.2. Quantification of Athletes’ Proprioceptive/Effective Shooting Volume—A Key for Scoring-Opportunity Identification

Scientifically, the quantification of a player’s attack volume would impartially show whether or not a ball should be counted as a chance of a goal. At the present time, there are few, if any, studies on the training manipulation and expansion of a player’s effective shooting volume. Within the current knowledge, the training effect on the volume depends on the learner’s spatial motor control ability, i.e., the proprioceptive competence, which is highly entrainable [48,49,50,51]. Up to now, this volume is still limited by the following SSTs in coaching practice [12,13,14]:

- Maximal instep kick (including curled kicks), characterized as kicks facing the goal, between balls, and ground balls;
- Headers, characterized as shots facing/side-facing the goal, between/side balls, and an air attack;
- Volleys, characterized as kicks facing/side-facing the goal, between/side balls, and an air attack below the hip.

Therefore, the current training system would under develop a player’s proprioceptive/effective shooting volume, reducing their possibilities to shoot and decreasing their goal-scoring chances.

The results of this study clearly demonstrated that the limited proprioceptive shooting volume is a result of the insufficient SSTs with which a player has been entrained. The results of Table 1 would suggest that talented athletes, e.g., Cristiano Ronaldo and Zlatan Ibrahimović (both winners of the Puskás Award and are able to perform a bicycle kick and a jumping side volley), have found ways to master unique acrobatic SSTs for increasing their proprioceptive/effective shooting volume by four times that of normally trained players. Evidently, these unusual SSTs contribute to enlarging their proprioceptive shooting volume. Furthermore, this study has revealed that, theoretically, the increased volume could potentially still be enlarged to seven or more times that of the “normally” practiced shooting volume (Table 1). The ramifications of our results are far-reaching. Even if less than half of these theoretical gains could be realized through the training of the underutilized “slices of the pie” from Figure 1, a player’s effective proprioceptive shooting volume could be doubled or tripled.

In short, to maximize scoring probabilities, it is essential to intensely expand the dimensions of the proprioceptive shooting volume, which is heavily influenced by the SSTs available to an athlete. The goals of Puskás nominees unveil that there are more than a dozen SSTs that should be considered as potential skills to maximize a player’s proprioceptive shooting volume. Based on FIFA criteria [22], these SSTs are undeniably repeatable; as such, they should be entrainable. What we need are scientific studies that demystify these SSTs and establish new and effective training regimes for developing these extraordinary SSTs among young and future players.

### 4.3. Focusing on Time in Space—The Nexus for Uniting Time Efficiency and Spatial Effectiveness

**Figure 5:**
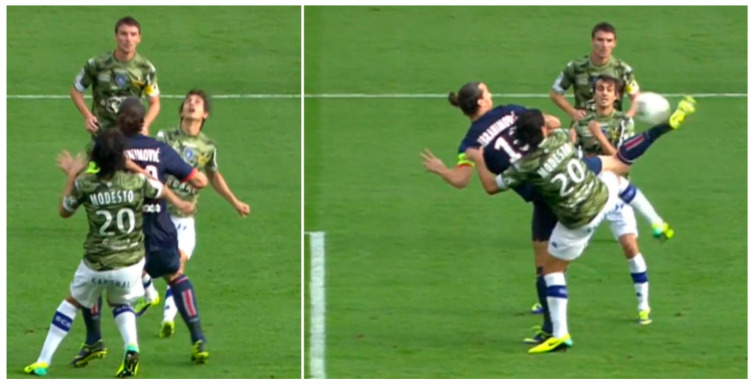
The scorpion kick, a nominated goal of FIFA Puskás Award 2014 performed by Ibrahimović (the figure was generated from the video of FIFA Puskás Award 2014).

The nexus for uniting the time efficiency and spatial effectiveness of players should be rooted in the development strategy of future SST training. The logical sequence would be:

- Firstly, master as many SSTs as possible through science-based SST training. This step would aim at enlarging the effective 3D dimensions of the proprioceptive shooting volume, i.e., increasing spatial effectiveness. This is the foundation for maximizing scoring possibilities;
- Secondly, entraining for decision ability in properly selecting an SST for an accurate attack by means of shooting various dynamic balls. This step would focus on temporal efficiency. Mastering more SSTs would allow a player to choose the proper SST for ensuring a zero-possession shot, regardless of the ball’s horizontal and vertical position as well as an attacker’s dynamic posture (Figure 1, Figure 2 and Figure 3);
- Lastly, further increase the decision ability with pre-judgements via practical training (e.g., game-like situations). This step would concentrate on making an achievable decision based on a reasonable pre-judgement during an in-game situation. After mastering various SSTs, a player would have more options to choose from when preparing an attack. How to plan an attack to preclude defensive players (for a clear shot) would be vital for shooting success. An excellent example is the scorpion kick (one of 2014 Puskás nominated goals [62]) performed by Ibrahimovic. During the game between Paris Saint-Germain and SC Bastia (Ligue 1/French), Ibrahimovic was running across the opponent’s penalty area (side-facing the goal) and received a chance on goal via his teammate’s airborne pass, which was falling into his proprioceptive shooting volume. Two defenders were applying a one-on-one defense on each of his sides (Figure 5 left) and attempting to defend the chance. Using his perfect pre-judgement ability, Ibrahimovic purposely moved further to let the ball fell behind him. This planned action precluded the defenders and created a free ball for a successful scorpion kick (Figure 5 right). Such a decision clearly needs the player to be able to perform a kick with a “beyond” and “airborne” ball.

Regrettably, scientific studies, as well as coaching practice, fall far behind in initiating the above training regime. Most of the identified SSTs (currently 43 in total [10]) cannot be systematically trained due to the lack of scientific investigation and understanding. As elaborated before, the SSTs overlooked by researchers and the current training regime are the airborne and/or acrobatic attack techniques, such as the scorpion kick, diving scorpion kick, jumping side volley, jumping turning kick, long-jump turning header, diving header, sliding kick, rabona kick, and more [10]. The fatal effect of the overlook is the decrease in time efficiency and spatial effectiveness.

One common characteristic of the SSTs overlooked in research and coaching practice is exceedingly complex, labeled as high-risk and low-reward [9]. These skills, for most athletes, cannot be learned without insightful guidance. It is time for researchers to conduct quantitative studies for (1) demystifying the complexity of the skills, (2) identifying the skills required for various ball positions and dynamic body postures, and (3) develop training programs for injury-free exercise, since the accurate performance of the airborne and/or acrobatic SSTs requires repetitive training.

This is the first study on the time and space aspects of SSTs. It is understandable that there are limitations associated with this study. There are two obvious ones. First, due to the unavailability of the 3D data of most SSTs, the current quantification on the spatial effectiveness can only be used as a reference for practitioners. More future studies are inevitably needed for reaching an accurate result. Second, there might be gender-based control-pattern variations. A quantitation of female athletes, as well as comparisons between the males and the females, must be conducted in the future studies.

Summarized above, the practical information for coaches and players is as follows: (1) the more SSTs an athlete can perform, the more goal chances he will have; and (2) the more airborne SSTs an athlete can master, the more efficient and effective his shooting attacks will be. Yet, merely relying on the aptness of an athlete to master these extraordinary SSTs would be hit or miss. Such an approach cannot be considered as a viable coaching strategy. A more science-structured learning, based on the re-conceptual organization of temporal-spatial aspects of SSTs should be developed. The development ought to firstly focus on improving players’ spatial awareness in terms of body orientation and ball spatial position and, subsequently, increase the temporal efficiency through practices of a one-touch-shot within this improved volume. In short, focusing on Time-in-Space would be the nexus for uniting time efficiency and spatial effectiveness in future SSTs learning and training.

## 5. Conclusions

Retrospectively, from a scientific standpoint, both temporal and spatial variables must be examined when evaluating soccer scoring opportunities. Unfortunately, field geography (i.e., the development of goal scoring opportunities) or game analysis in elite soccer tends to dominate the attention of researchers and practitioners and consideration of the player-centered temporal-spatial aspects of SSTs is mostly neglected.

Goal scoring research fails to address the time and space related to SSTs. Space certainly consists of more than mere field geography. A player’s trained SSTs influence both scoring opportunity identification and the dimensions of his/her attack space. The development of novel training programs should, first, focus on the increase of the proprioceptive shooting volume through mastering as many SSTs as possible; then, it should concentrate on the training of selecting a proper SST to reach one-touch-shots within the enlarged attack volume, i.e., focusing on time in space.

The current study reveals that a player has to learn airborne and acrobatic SSTs in order to increase his/her spatial effectiveness, as well as the temporal efficiency of shooting. Therefore, scientific studies are indisputably needed to demystify the complexity of these skills for developing their learning and training.

The great attraction of soccer for millions of fans is the goal. Various techniques for scoring goals are sources of excitement. More frequent use of airborne and acrobatic SSTs for goals can only enhance the excitement of the game. Therefore, this new theoretical framework would bring more excitement by promoting novel studies and developing innovative training programs for learning and practicing various SSTs.

## Figures and Tables

**Table 1:** The comparison of the proprioceptive/effective shooting volume from the current practice (100%) to the player-centered temporal-spatial training of airborne/acrobatic SSTs.

Trained through Currently Practice	Achieved by Talented Elite Athletes	Theoretical Potential
100%	~400%	~700%

## Data Availability

Not applicable.
